# Complexity Measures: Open Questions and Novel Opportunities in the Automatic Design and Analysis of Robot Swarms

**DOI:** 10.3389/frobt.2019.00130

**Published:** 2019-11-26

**Authors:** Andrea Roli, Antoine Ligot, Mauro Birattari

**Affiliations:** ^1^Department of Computer Science and Engineering, Campus of Cesena, Alma Mater Studiorum Università di Bologna, Bologna, Italy; ^2^IRIDIA, Université libre de Bruxelles, Brussels, Belgium

**Keywords:** complexity measures, information theory, swarm robotics, evolutionary robotics, automatic design

## Abstract

Complexity measures and information theory metrics in general have recently been attracting the interest of multi-agent and robotics communities, owing to their capability of capturing relevant features of robot behaviors, while abstracting from implementation details. We believe that theories and tools from complex systems science and information theory may be fruitfully applied in the near future to support the automatic design of robot swarms and the analysis of their dynamics. In this paper we discuss opportunities and open questions in this scenario.

## 1. Introduction

Metrics that quantify the complexity of a system and measure information processing are used in a wide range of scientific areas, including neuroscience, physics, and computer science. In the scientific literature, the word *complexity* is overloaded, as it may refer to the amount of effort needed to describe a system, or to create it, or also to quantify its structure both in terms of components and dynamical relations among its parts. For example, let us consider a swarm of robots: we may ask what is the complexity of a function describing the overall behavior of the swarm, or what is the complexity of the problem of optimally assigning tasks to the robots, or what is the complexity of each of the tasks. These objectives require different measures, each addressing a specific question. As a consequence, there is no unique and all-encompassing complexity measure: a plethora of metrics are available. Most come from information theory, which abstracts from specific system's details and focuses on information processing. While notable results have been attained, we believe that the potential of these methods has still to be fully exploited in the automatic design of robot swarms and in the analysis of their behaviors.

In automatic design methods, the design problem is cast into an optimization problem that is solved either off-line or on-line, i.e., either before the swarm is deployed in its target environment or while the swarm is operating in it. A prominent example of automatic design is evolutionary robotics (ER), where the control software—typically an artificial neural network (ANN)—is optimized by means of an evolutionary algorithm (Nolfi and Floreano, 2000). A number of alternative methods depart from the classical ER by employing control software architectures other than ANNs and/or optimization techniques other than evolutionary computation (Watson et al., 2002; Hecker et al., 2012; Francesca et al., 2014; Gauci et al., 2014). A review of the main studies on automatic design of robot swarms—both off-line and on-line—is provided by Francesca and Birattari (2016).

The aim of this paper is to outline what we think are the most important open questions and to describe opportunities to use complexity measures for supporting the automatic design of swarms of robots and the analysis of their behaviors. In section 2, we provide an introduction to complexity measures. In section 3, we highlight the main contributions to the robotics field. In section 4, we illustrate our perspective and outline relevant open questions.

## 2. A Capsule Introduction to Complexity Measures

The notion of complexity is multifaceted. If, by the term “complex,” one means “difficult to predict,” then a suitable metric is provided by *information theory* with *Shannon entropy* (Shannon, 1948). Let us consider a simple system of which we observe the state at a given time. The observations can be modeled as a random variable *X*, which can assume values from a finite and discrete domain X. If the observation is x∈X, which has a probability *P*(*x*), then the amount of information carried by the observation of *x* is defined as $\frac{1}{\log P\left(x\right)}=-\log P\left(x\right)$^1^. Shannon entropy is defined as the expected value of the information of all symbols: $H\left(X\right)=-\underset{x\in X}{\sum }P\left(x\right)\log P\left(x\right)$. Intuitively, *H*(*X*) measures the amount of surprise—or, equivalently, the lack of knowledge—about the system; we may also observe that Shannon entropy measures the degree of disorder in a system or process. Many complexity measures are based on Shannon entropy. For example, the reciprocal influence between two parts of a system can be estimated by computing their *mutual information*, defined as *I*(*X*; *Y*) = *H*(*X*) + *H*(*Y*) − *H*(*X, Y*), where *H*(*X, Y*) is the joint entropy of the variables *X* and *Y*, defined on the basis of the joint probability *P*(*x, y*). *I*(*X*; *Y*) provides a measure of the information we can gain on a variable, by observing the other. Information-theoretic metrics are currently widely applied, as they have the property of being model independent and able to capture non-linear relations. In practice, probabilities are usually estimated through the observed frequencies.

When the objective is to measure the complexity of the description of a system, then *algorithmic complexity* may be used, as proposed by Kolmogorov (1965): the complexity of a string of symbols is defined as the length of the shortest program producing it. This measure is not computable in general, but approximations are available, such as the ones based on compression algorithms (Lempel and Ziv, 1976). Shannon entropy and Kolmogorov complexity are conceptually different (Teixeira et al., 2011). The former measures the average uncertainty of a random variable *X*, and so it estimates the difficulty of predicting the next symbol of a sequence received from a source. Conversely, Kolmogorov complexity measures the length of the minimal (algorithmic) description of a given sequence of symbols σ, therefore it estimates the difficulty of describing or reconstructing the sequence. However, they both capture the notion of compressibility of a signal and, in particular, they are null when *X* (resp. σ) is constant and maximal when *X* (resp. σ) is random.

Kolmogorov complexity also provides a theoretical framework for the principle known as *Occam's razor* that states that among all the possible explanations of a set of data, the simplest one is preferable. A similar argument supports the notion of *stochastic complexity*, proposed by Rissanen (1986), which is the shortest description of the data with respect to a given probabilistic model.

The term “complex” is often used for capturing the notion of structure or pattern observed in data or in the dynamics of a system, once random elements are discarded. This concept is also related to the extent to which correlations distribute across the parts of the system observed (Grassberger, 1986a). The intuition is that high complexity should be associated to conditions characterized by a mixture of order and disorder, structure and randomness, easily predictable dynamics and novelty. Along this line, several measures have been proposed (Grassberger, 1986a; Lindgren and Nordahl, 1988; Li, 1991; Crutchfield, 1994; Gell-Mann and Lloyd, 1996; Shalizi and Crutchfield, 2001). A survey on complexity metrics is out of the scope of this contribution and we refer the interested reader to prominent works on the subject (Grassberger, 1986a; Lindgren and Nordahl, 1988; Badii and Politi, 1999; Lloyd, 2001; Prokopenko et al., 2009; Lizier, 2013; Moore et al., 2018; Thurner et al., 2018; Valentini et al., 2018).

## 3. Complexity Measures in Robotics

A possibility for using information theory in robotics is enabled by the notion of sensory-motor coordination (Pfeifer and Scheier, 1997) which emphasizes the role of the loop between sensors and actuators in robots performing cognitive tasks. Sensory-motor coordination models can be described in terms of dynamical systems and control theory, which are suitable for analyses based on information theory. More specifically, the sensory-motor loop expresses both the effect that sensors have on actuators and the effect that actuators have on sensors. The former is mediated by the robot's control software; the latter by the environment. Information-theoretic measures can be used to study some properties of system dynamics (Islam and Murase, 2005; Lizier, 2013; Beer and Williams, 2015; Da Rold, 2018) and to characterize the information flow in the sensory-motor loop (Lungarella and Pfeifer, 2001; Lungarella et al., 2005; Lungarella and Sporns, 2006; Ay and Zahedi, 2014). Notably, this approach makes it possible to quantitatively study the relation between the robot and the environment (Beer, 1995, 2008; Smithers, 1995; Tarapore et al., 2004, 2006; Nehmzow, 2008; Schmidt et al., 2013; Butail et al., 2014; Izquierdo et al., 2015), which is fundamental in embodied systems (Pfeifer and Scheier, 2001). A sound and thorough treatise on the dynamics emerging from the interaction among robot, control program and environment is provided by Nehmzow (2008).

Information-theoretic measures typically used in these contexts are mainly based on Shannon entropy (Cover and Thomas, 2012) and range from *mutual information* (Lindgren, 2014) and *transfer entropy* (Schreiber, 2000) to *predictive information* (Grassberger, 1986b; Crutchfield and Young, 1989; Bialek et al., 2001; Martius et al., 2013). Predictive information (PI) has been successfully used to quantify properties of emergent behaviors and as an objective function for designing robots able to show the so-called *self-organization dynamics* (Der et al., 2008; Martius and Olbrich, 2015). The PI of a system is computed by extracting a time series from a set of variables, e.g., the robot's sensor readings. The PI of the system is then defined as the mutual information of the future and the past within the time series. If the robot's behavior is such that features of the environment (e.g., a light gradient) are exploited to achieve the robot's goals (e.g., phototaxis), then the sensory-motor loop tends to produce coordinated patterns. These patterns are captured by high values of PI computed across sensor time series. Conversely, maximizing PI produces self-organized behaviors (Ay et al., 2008).

To the best of our knowledge, Odagiri et al. (1998) were the first to study the complexity of an ANN controlling a robot and to highlight its correlation with the complexity of the environment in which it is evolved^2^. In their work, the ANN controlling a Khepera robot is evolved in different environments characterized by different levels of complexity (obtained by construction) and the complexity of the ANN is evaluated in terms of non-zero weights in the network. While this way of estimating ANN complexity is common in machine learning and is not based on information theory, this paper provides a clear statement of the problem. Results show that the ANN complexity is correlated with the complexity of the environment and the authors suggest to use this metric to estimate the complexity of the environment “as seen by the robot.” A similar work achieving analogous conclusions has been proposed by Capi (2007). Yang and Anderson (2011) address the same problem by evolving artificial agents that have to reach a target cell in a grid with obstacles; robots are guided by means of a depth search algorithm. The authors propose a metric for estimating environmental complexity based on entropy and compressibility of the grid in which the agents move. A linear regression model on these two variables is then inferred and used to estimate the average number of steps required by the agent to reach the target.

As different complexity measures capture different features of a system, one should aim at producing a *complexity fingerprint* by computing several metrics, rather than identifying a single metric able to summarize all the relevant properties related to complexity (Roli et al., 2018). Teo and Abbass (2005) address this issue by proposing a multidimensional complexity measure, consisting of several different metrics by means of which a partial ordering can be defined.

A prominent question in natural and artificial evolution is whether complexity increases over generations and what is its relationship with fitness. A recent work (Joshi et al., 2013) addresses this issue in simulation by evolving artificial agents controlled by extended ANNs. The metrics used in this research are mutual information, predictive information and also *integrated information* (Balduzzi and Tononi, 2008). This latter metric has been proposed with the aim of capturing to what extent a system is able to integrate information coming from the environment. Agents controlled by a network of stochastic transition functions have been subject to artificial evolution with the goal of finding the exit of a maze in the shortest time. The outcome of the research is that complexity is positively correlated with fitness. More precisely, the minimal complexity among all the evolved ANN corresponding to any one fitness value is a quantity increasing with fitness. A measure of behavioral diversity in a group of robots has been proposed by Balch (2000) who defines a metric based on hierarchical clustering of behaviors across the group of robots. This information-theoretic approach enables us to quantitatively correlate the heterogeneity of the group of robots with performance.

Information-theoretic measures have also been used as task-agnostic merit factors for the design of coordinated behaviors in evolutionary robotics^3^. An example of this approach is the achievement of a coordinated behavior by maximizing the average mutual information between all pairs of robot motors (Olsson et al., 2005; Sporns and Lungarella, 2006; Salge and Polani, 2011; Sperati et al., 2014). The aim of using information-theoretic measures in this setting is to bias evolution toward robot control software that enables the robots to attain some useful emergent property, which can be exploited for the specific task at hand^4^. Of similar spirit are the works of Martius et al. (2013) and Ay et al. (2008), which present a principled approach to derive control software on the basis of integrated information for attaining self-organized and explorative behaviors. The coordination of a group of robots is the focus of work by Capdepuy et al. (2007), in which the multi-robot case is subjected to an information-theoretic analysis. Finally, we mention the work by Klyubin et al. (2008), where a new metric called *empowerment* is proposed with the aim of guiding evolution to producing robots that, all things being equal, choose actions that maximize the number of their future possible actions.

## 4. Open Questions and Opportunities for Future Research

Recent results attained by using information-theoretic and complexity measures in analysing and designing robot behaviors motivate further investigations. Some questions are still open and we believe that addressing them will improve swarm robotics, in particular. Here we focus on a specific subject that we think has still to be thoroughly addressed: the relation between complexity of individual robot, swarm and environment. As the observed behavior of a robot is the result of the interplay of control software, robot's physical features and environment, the complexity of an individual robot does not necessarily correspond to the complexity of its control software. Complex control software might produce simple behaviors because the robot cannot exploit any significant property of the environment or because the environment is overconstrained; conversely, simple control software may generate rather complex behaviors by exerting simple interactions in a complex environment. This, in turn, poses the question as to what extent the complexity of the environment can be assessed: is it possible to measure environment complexity without referring to a specific robot platform? If not, are there general approaches to assess relative complexity? Should one abandon the idea of measuring the complexity of the two entities separately and measure the complexity of the compound instead? The situation is of course more complicated in the case of robot swarms, as interactions among robots produce an emerging behavior at a higher level. The relation between the complexity of individual robot and the swarm is again to be studied: is it in general possible to attain any complexity level in the swarm independently of the individual complexity by operating on the interactions? If not, are there bounds on the complexity that can be computed? Concepts and methods from the physics of collective systems and statistical physics (Nicolis and Prigogine, 1977; Binney et al., 1992; Kauffman, 1993; Bar-Yam, 1997; Badii and Politi, 1999) are likely to provide useful tools for addressing these questions, especially for what concerns the relation between micro and macro levels, i.e., between robots and swarm. Nevertheless, the hypothesis that robots behave as particles is seldom verified and new advancements of these theories should be developed to properly address these questions. Furthermore, we may ask if meso-levels appear as intermediate structures between micro and macro levels, as in the case of the so-called *sandwiched emergence* (Lane, 2006); these meso-levels have indeed a bidirectional effect as they influence both upper and lower levels.

We believe that addressing these questions would lead to important improvements to the automatic design of robot swarms. Swarm robotics is a promising approach to coordinating large groups of robots (Dorigo et al., 2014; Yang et al., 2018), which has already attracted the attention of the wider scientific community (e.g., see Rubenstein et al., 2014; Werfel et al., 2014; Garattoni and Birattari, 2018; Slavkov et al., 2018; Yu et al., 2018; Li et al., 2019; Xie et al., 2019). As observed by Brambilla et al. (2013), an engineering approach to the development of robot swarms is still in its infancy (Winfield et al., 2005; Brambilla et al., 2012, 2015; Francesca et al., 2015; Francesca and Birattari, 2016; Khaluf et al., 2016). In automatic design, a space of possible design instances is explored by an optimization algorithm with the goal of maximizing an appropriate mission-specific performance measure. In the off-line case, the performance of candidate design instances explored by the optimization algorithm is assessed via computer simulations (Birattari et al., 2019). One of the main issues to be addressed in this case is the discrepancy between simulation and reality. This discrepancy—usually named the *reality gap*—is often the reason for performance drops when control software developed in simulation is deployed on real robots. Recently, Birattari et al. (2016) show that the reality gap is the cause of what has been named *overdesign*: in an off-line automatic design process, the performance in simulation steadily increases with design effort (e.g., iterations of the optimization algorithm), while the one in reality increases up to a certain level and then starts decreasing. Furthermore, the performance drop due to the reality gap is a relative problem: different design methods are affected to a different extent by the same difference between simulation and reality (Francesca et al., 2014) and a rank inversion can be observed: on the same mission, the control software produced by design method *A* might perform better than the one produced by design method *B* when they are evaluated in simulation; while it could be the other way around when the control software is ported to the robots. Overdesign and eventually performance drop and rank inversion have a statistical interpretation in terms of model identification: the choice of the structure of a statistical model implies—even implicitly—a trade-off between *bias* and *variance*, which represent two kinds of error affecting an estimator. Models of a relatively simple structure are characterized by high bias and tend to under-fit data, whilst those of a relatively complex ones are affected by a large variance and tend to overfit (Geman et al., 1992). As it has been recently shown, performance drop and rank inversion may be observed also in a simulation-only experiments (Ligot and Birattari, 2018, 2019): that is, when behaviors are designed using a simulation model and tested using another one. This indicates that performance drop and rank inversion should not be ascribed to some unique relationship between reality and the simulation environment used in the design process, but rather to an intrinsic characteristic of a design method—i.e., the complexity of the control software produced: high complexity exposes a method to the risk of overfitting the simulation environment used in the design process. Overfitting is commonly addressed by invoking the *parsimony principle*, stating that a model should be as simple as possible. In fact, this principle can be seen as a statistical formalization of the Occam's razor, usually expressed as “shave away all that is unnecessary” or “everything should be made as simple as possible, but not simpler” (Burnham and Anderson, 2002).

In the light of this last consideration, we believe that a principled estimation of the complexities of swarm, individual robots and environment may help address this issue by suggesting the best levels of complexity to attain in the design. Contrary to some established fields like statistics and machine learning in which the scientific community has agreed on the adoption of some complexity measures—e.g., and Akaike's information criterion (Akaike, 1973) and Vapnik-Chervonenkis dimension (Vapnik, 1995)—the swarm robotics community has not yet reached consensus on any complexity measure.

**Figure 1:**
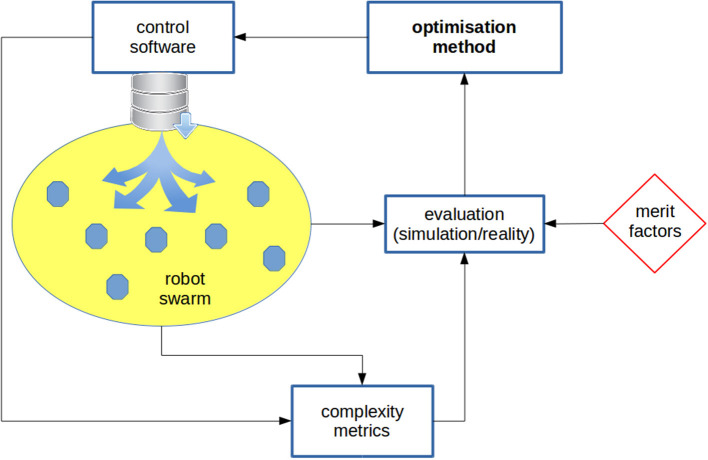
Generic design cycle: the control software is deployed onto the robots, which are then evaluated; on the basis of the evaluation, some parameters of the control software are changed by means of an optimization method. Complexity metrics are fed into the evaluation block. Off-line design: robots are evaluated in simulation. The metrics can be used as a regularization component of the fitness function used in the evaluation block: besides a fitness component accounting for the performance of the swarm on the specific task, a further component can be added to penalize (i) individual robots or (ii) swarms that exhibit a complexity level not complying with the requirements. In the first case, the average *predictive information* (PI) of the robots can be taken, whilst in the second case the *average pairwise mutual information* can be used (see Figure 2). Alternatively, a multi-objective approach can be chosen, in which the swarm performance and the complexity metric are the two criteria considered in the optimization process. In this latter case, the user can choose a personal trade-off between performance and complexity. Mixed off-line and on-line design: an off-line design phase is first run, in which the complexity of the swarm (or the individuals, or both) is maximized. Subsequently, an on-line design process takes place for tuning the control software parameters with the goal of specializing the swarm to the task at hand and to keep the complexity in a given range.

**Figure 2:**
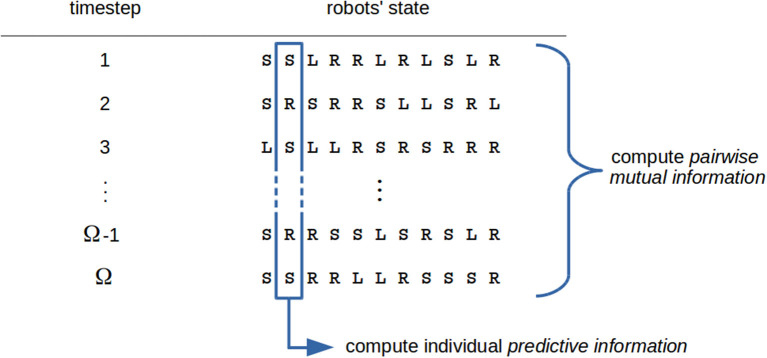
An example of the evaluation of complexity metrics. We suppose that robots are controlled by a probabilistic finite state automaton, hence the swarm state is given by a tuple composed of symbols from a finite alphabet, each representing one state in the automaton (this choice is not a limitation, as it is possible to compute complexity metrics for any choice of the variables representing robots' state). Individual metrics can be computed by considering the time sequence of states for each robot, while global swarm measures are a function of the whole swarm state (or a portion of it). In the figure, the *predictive information* (PI) of each robot can be computed on the basis of the time sequence of robot states; conversely, as a swarm measure the *average pairwise mutual information* can be computed. Indeed, high values of this metric are expected to favor global coordinated behaviors (Sperati et al., 2014).

In a futuristic scenario, we imagine the possibility of measuring the complexity of a swarm robotics task and designing the control software accordingly. Besides the questions already stated, this perspective poses further issues, as measuring the complexity of a task is a non-trivial problem and in general it depends on the robot's model. We believe that information theory may provide a suitable framework for addressing these questions because it makes it possible to abstract from implementation details and focus on interactions and dynamics related to information processing. In Figures 1 and 2, an example of the use of complexity measures inside the automatic design flow is sketched.

Besides the open questions already mentioned, we also envision room for improvement in the use of task-agnostic merit factors in automatic design of robot swarms. While some results have been attained (Sperati et al., 2008; Der and Martius, 2012; Mouret and Doncieux, 2012), we believe that the potential of this approach has still to be fully expressed. For example, we expect that combining complexity measures with task-specific objective functions—e.g., in a multi-objective framework—may lead to swarms characterized by fast re-calibration in case of environmental or task changes. In the longer term, we envision the possibility of tuning the complexity of an adaptive swarm depending on the—possibly changing—requirements. For example, highly complex control software may be employed with its complexity controlled during the design process or even dynamically during operation. A viable way to attain this could be either *a priori* designing the control software instances so as to tune their complexity by means of specific variables acting on their structure and parameters (internal regulation) or by properly calibrating the interactions between robot and environment, and between the robots of the swarm. This last kind of control (external regulation) may be achieved, for example, by properly choosing type and properties of environmental features (i.e., by tuning the robots' environmental niche) or by selecting which sensors and actuators the robots can use.

Apart from few preliminary investigations (Roli et al., 2013, 2015, 2018), there is still much room for application of information-theoretic and complexity measures in the analysis of robot behavior. Besides the use of such metrics during the design process (e.g., to assessing the complexity of the swarm or the individual robots), we envisage a real-world scenario in which the complexity level of a robot swarm is monitored in order to detect specific phases of its behavior (e.g., when a decision has to be collectively taken) and possible failures—e.g., when a large discrepancy between the expected and actual complexity of the swarm is observed it might be the case that some malfunctioning dynamics is taking place.

## Author Contributions

AR coordinated the paper and drafted a preliminary version of the manuscript. All authors equally contributed to the ideas and the writing.

### Conflict of Interest

The authors declare that the research was conducted in the absence of any commercial or financial relationships that could be construed as a potential conflict of interest.

## Footnotes

^1^Usually, the logarithms are taken to the base 2, so as to express information in terms of bits.

^2^The generation of environments with different degrees of complexity for testing or evolutionary purposes is not new. E.g., see Stirling et al. (2010).

^3^The use of task-independent objective functions in artificial evolution is not a new idea—e.g., see Lehman and Stanley (2011). However, in this paper we are concerned only with approaches related to complexity and information-theoretic measures.

^4^A similar approach is that of *guided self-organization* (Prokopenko, 2014).
